# Statistical Interior Tomography via L_1_ Norm Dictionary Learning without Assuming an Object Support

**DOI:** 10.3390/tomography8050186

**Published:** 2022-09-02

**Authors:** Junfeng Wu, Xiaofeng Wang, Xuanqin Mou

**Affiliations:** 1 Department of Applied Mathematics, Xi’an University of Technology, Xi’an 710048, China; 2 The Institute of Image Processing and Pattern Recognition, Xi’an Jiaotong University, Xi’an 710049, China

**Keywords:** dictionary learning, direct current component, interior tomography, statistical iterative reconstruction

## Abstract

Interior tomography of X-ray computed tomography (CT) has many advantages, such as a lower radiation dose and lower detector hardware cost compared to traditional CT. However, this imaging technique only uses the projection data passing through the region of interest (ROI) for imaging; accordingly, the projection data are truncated at both ends of the detector, so the traditional analytical reconstruction algorithm cannot satisfy the demand of clinical diagnosis. To solve the above limitations, in this paper we propose a high-quality statistical iterative reconstruction algorithm that uses the zeroth-order image moment as novel prior knowledge; the zeroth-order image moment can be estimated in the projection domain using the Helgason–Ludwig consistency condition. Then, the L_1_norm of sparse representation, in terms of dictionary learning, and the zeroth-order image moment constraints are incorporated into the statistical iterative reconstruction framework to construct an objective function. Finally, the objective function is minimized using an alternating minimization iterative algorithm. The chest CT image simulated and CT real data experimental results demonstrate that the proposed approach can remove shift artifacts effectively and has superior performance in removing noise and persevering fine structures than the total variation (TV)-based approach.

## 1. Introduction

The Interior problem reconstructs a region of interest (ROI) inside a patient or an object from data along X-ray paths through the ROI [1]. This ROI reconstruction problem does not have a unique solution in an unconstrained image space, even if we know the object support (OS) exactly [2]. As a result, reconstructions with filtered backprojection (FBP) suffer from a Direct Component(DC) shift and low-frequency artifacts, which make it difficult to meet the clinical diagnostic needs of physicians.

An X-ray is harmful to the human body. Hence, it is highly desirable to perform interior reconstruction for radiation dose reduction. Over the last few decades, a variety of ROI image reconstruction algorithms, which can improve the quality of reconstructed ROIs by incorporating smart prior knowledge into the image reconstruction process, have been developed. In particular, a theoretically exact local reconstruction methodology, which is referred to as interior tomography, has been proved and developed. Kudo H. and Ye YB have proven that if a sub-region in an internal ROI is known, the ROI can be reconstructed using differentiated backprojection (DBP) projection on a convex set (POCS) technique [3,4,5]. Subsequently, a singular value decomposition (SVD)-based DBP method has been developed for interior tomography [6,7]. Jin X et al. improved a novel continuous SVD method for interior reconstruction, assuming a known sub-region [8]. However, in many important applications, such as perfusion cardiac CT and micro-CT, a sub-region is not always available.

Inspired by compressed sensing theory, researchers have proposed an accurate reconstruction algorithm which incorporates sparse prior information of ROI into an iterative reconstruction model. For example, Yu et al. showed that if ROI can be expressed as a segmented constant function, the algebraic iterative reconstruction algorithm with total variation (TV) sparse constraints can accurately reconstruct ROI images [9,10]. Yang et al. proposed that if ROI is a piecewise polynomial function, then ROI can be accurately reconstructed by minimizing the high-order TV sparsity constraint [11]. With the development of compressed sensing theory, many new sparse priors have been introduced to improve the quality of reconstructed ROI. Esther Klann et al. developed an algorithm for interior tomography of a piecewise constant function using a Haar wavelet basis [12]. John Paul Ward et al. proved that the exact reconstruction of ROI is guaranteed using a one-dimensional generalized total variation seminorm penalty [13,14]. Subsequently, Liu et al. employed the curvelet transform coefficients to regularize the interior problem and obtained a curvelet frame-based regularization method for interior tomography [15]. Zhao et al. applied the Mumford–Shah-TV regularization method to the interior tomography and developed an algorithm based on split Bregman and OS-SART iterations [16]. Tatiana A. Bubba et al. proposed a nonsmooth regularization approach based on shearlets for ROI tomography [17]. Nevertheless, all of the above algorithms exploit the sparse property of the image but ignore the fact that the projection data usually contain noise during a real CT scan. Considering the noise characteristics of the projection data, Xu et al. proposed a statistical interior tomography method with a TV sparsity constraint [18]. However, the ROI images reconstructed with TV regularization may lose some detailed features and contain blocky artifacts in the case of noisy projections.

One crucial issue in interior tomography is that intensities drop around the peripheral region of a ROI. When a priori information does not exist or is not sufficiently strong, image intensity could deviate dramatically from the truth near the boundary of the ROI. There are a number of ways to suppress this problem by introducing additional information about the object to be reconstructed. Currently, an effective means is to enforce object support (OS). However, this is not always available in practical scenarios. Hence, we hypothesize that the zeroth-order image moment is a surrogate, which can be easily measured in the projection domain.

Sparse representation and dictionary learning (DL) have achieved great success in the medical imaging community, such as in low-dose image reconstruction [19,20], limited-angle CT reconstruction [21], spectral CT [22], etc. The extensive experimental results have shown that the DL-based regularization term is superior to the TV-based candidate in terms of preserving image details and removing artifacts. Encouraged by the above findings, here, we introduce the DL and zeroth-order image moment into the statistical iterative reconstruction (SIR) framework for interior tomography to further lower the radiation dose. The zeroth-order image moment can be estimated in the projection domain using the Helgason–Ludwig consistency condition (HLCC). An alternating minimization algorithm is developed to optimize the objective function.

The novelties of the proposed method are as follows. Firstly, an l1-normdictionary learning penalty is introduced into the SIR framework for the ROI image reconstruction to obtain a reconstruction performance that is superior to the existing TV penalty. Secondly, most CS-based interior tomography methods assume that the object support is known before the reconstruction of the proposed SIRDL + HL method using direct current priors which can be estimated using projection data and the Helgason–Ludwig consistency condition. Thirdly, an alternating minimization algorithm is developed to minimize the associated objective function, transforming the image reconstruction problem into a sparse coding sub-problem and an image updating sub-problem. To accelerate the convergence speed of the proposed alternating minimization algorithm, an order subset strategy is applied during the iteration process.

The rest of this paper is organized as follows. In Section 2, we first briefly review related mathematical theories and then establish the proposed reconstruction framework. In Section 3, both the numerical simulation and Sheep lung real CT projection experiments are performed to evaluate the proposed method. In Section 4, we discuss the results and conclude the paper.

## 2. Materials and Methods

### 2.1. Statistical Iterative Reconstruction

The transmission X-ray CT can be described in a discrete form using the following statistical model:

(1) $$y_{i}∼\mathrm{Poisson}(b_{i}\;\exp(-p_{i})),\;i=1,\ldots ,I,$$

where $y={\left(y_{1},\ldots ,y_{I}\right)}^{T}$ is a vector representing the raw detector measurements, where the corresponding blank scan $b={\left(b_{1},\ldots b_{I}\right)}^{T}$, $p_{i}=\sum_{j=1}^{J}a_{ij}\mu_{j}={\left[A\mu \right]}_{i}$ is the projection data that measure the integral of the X-ray linear attenuation coefficients; $\mu ={\left(\mu_{1},\cdots ,\mu_{J}\right)}^{T}$ is a vector representing the attenuation coefficients of the imaged object; $A=\left\{a_{ij}\right\}$ is the I×J system matrix that models the imaging system; the element $a_{ij}$ of the system matrix represents the length of the intersection between the *i*th projection path and the *j*th pixel, which is determined by the focal spot position and the detector pixel position and the symbol T denotes the transpose operator.

SIR is obtained by minimizing the following cost function:

(2) $$\Phi (\mu )=\left\{\sum_{i=1}^{I}\frac{w_{i}}{2}{\left({\left[A\mu \right]}_{i}-p_{i}\right)}^{2}+\beta U(\mu )\right\}\;$$

where $w_{i}$ respects the maximum likelihood estimated by the inverse of the variance of the projection measurement; U(μ) is known as the regularization term that presents prior knowledge of the image object and β is the regularization parameter that adjusts the balance between the data-fitting term and the regularization term.

### 2.2. L_1_Norm Sparse Representation Based on Learned Dictionary

Leta binary matrix $R_{s}=\left\{r_{nj}^{s}\right\}\in R^{N\times J}$ be an operator that extracts the s-th patch of μ. $R_{s}\mu$ is the vector representation of a square 2D image patch of $\sqrt{N}\times \sqrt{N}$ pixels. $D\in R^{N\times M}$ is a dictionary of *M* atoms represented in columns $d_{k}\in R^{N}$. Then, the sparse representation of $R_{s}\mu$ with D is to find a sparse vector $\alpha =\left[\alpha_{1};\ldots ;\alpha_{M}\right]$ (i.e., most of the coefficients in α are zero or insignificant) such that $R_{s}\mu \approx D\alpha$. The sparse vector α can be obtained by solving the following minimization problem:

(3) $$\arg\underset{\alpha }{\min}\left\{{\left\|R_{s}\mu -D\alpha \right\|}_{2}^{2}+\lambda {\left\|\alpha \right\|}_{1}\right\}\;$$

where λ is a regularization parameter.

A key component of sparse representation modeling is the choice of dictionary. In the majority of cases of good dictionary learning, the training patches are usually extracted from the high-quality sample images of the same style to become the reconstructed image. Given a batch of training patches $X=\left[x_{1},\ldots x_{S}\right]\in R^{N\times S}$, the target of dictionary learning is to find a dictionary that makes each patch in the training set sparsely represented by the atoms of the dictionary; that is, to jointly optimize the dictionary D and the representation coefficient matrix $\alpha =\left[\alpha_{1},\ldots \alpha_{S}\right]$ such that $x_{i}\approx D\alpha_{i}$ and ${\left\|\alpha_{i}\right\|}_{1}\leq T$. This can be expressed by the following minimization problem:

(4) $$\arg\underset{\alpha ,D}{\min}\left\{{\left\|X-D\alpha \right\|}_{2}^{2}+\lambda {\left\|\alpha \right\|}_{1}\right\}\;$$

A fast online learning algorithm has been developed to solve the above dictionary learning problem [23], which is an iterative optimization method that alternates between sparse coding of the image patch set based on the current dictionary and updating the dictionary D. An optimization toolbox named Sparse Modeling Software has been distributed on the internet to solve the above dictionary learning problem [24]. In this paper, we apply this toolbox to train a dictionary.

### 2.3. Direct Current Priors

The HLCC plays an important role in image reconstruction from the imperfect projection data [25] (e.g., due to noise, motion and truncation), since these projections no longer satisfy the HL conditions. The zeroth-order HLCC applied to the equi-angular fan-beam geometry can be expressed in the following equation [26]:

(5) $$\int_{-\pi /2}^{+\pi /2}p(\xi ,\psi +\xi -\pi /2)E\cos\xi d\xi =m_{0,0}\;$$

where E is the distance from an X-ray source to the rotation center and $m_{0,0}$ is the zeroth-order image moment, namely the sum of the image pixel values (which is the so-called direct current (DC) component). The variables ξ and ψ are the angle of an X-ray relative to the central X-ray and a projection view angle, respectively. After computing the DC value, denoted as C in the following, of the image to be reconstructed using Equation (5), we can incorporate the difference between the measured/assumed and reconstructed results into the objective function in Equation (2). In this study, we consider C as a prior estimated from clinical CT images, which can also be directly measured with one or more complete fan-beam view.

### 2.4. Optimization of SIRDL + HL Method

In this paper, we introduced the sparse representation based on L_1_norm dictionary learning and direct current priors into the SIR framework. This is different from most CS-based interior tomography methods, assuming that the object support is known before reconstruction. We proposed using direct current priors which can be estimated using projection data and HLCC. The joint regularization model based on L_1_ norm dictionary learning and HLCC, termed as SIRDL + HL, can be formulated as:

(6) $$\underset{\mu ,\alpha_{s}}{\min}\left\{\sum_{i=1}^{I}\frac{w_{i}}{2}{\left({\left[A\mu \right]}_{i}-p_{i}\right)}^{2}\left.+\beta \sum_{s=1}^{S}\left({\left\|R_{s}\mu -D\alpha_{s}\right\|}_{2}^{2}+\lambda {\left\|\alpha_{s}\right\|}_{1}\right)+\gamma {\left\|\sum_{j=1}^{J}\mu_{j}-C\right\|}_{2}^{2}\right\}\right.\;$$

where β is the regularization parameter that balances the data-fitting term and L_1_ norm sparse representation based on the learned dictionary term, and γ is the regularization parameter that balances the data-fitting term and the direct current prior regularization term. The proposed objective function can be efficiently solved by an alternating minimization algorithm. Mathematically, Equation (6) contains the following two sub-problems, which can be solved in an alternation manner of the two following sub-problems.

Sub-problem A:

(7) $$\underset{\alpha }{\min}\left\{\sum_{s=1}^{S}\left({\left\|R_{s}\mu -D\alpha_{s}\right\|}_{2}^{2}+\lambda {\left\|\alpha_{s}\right\|}_{1}\right)\right\}\;$$

The sub-problem A is called sparse representation, which is closely related to one of the following convex constrained optimization problems:

(8) $$\underset{\alpha }{\min}{\left\|\alpha_{s}\right\|}_{1}\;s.t.\;{\left\|R_{s}\mu -D\alpha_{s}\right\|}_{2}^{2}\leq \varepsilon \;$$

where ε is a nonnegative real parameter. In this paper, the LASSO algorithm [23] is applied to solve Equation (8).

Sub-Problem B:

(9) $$\underset{\mu }{\min}\left\{\sum_{i=1}^{I}\frac{w_{i}}{2}{\left({\left[A\mu \right]}_{i}-p_{i}\right)}^{2}\left.+\beta \sum_{s=1}^{S}{\left\|R_{s}\mu -D\alpha_{s}\right\|}_{2}^{2}+\gamma {\left\|\sum_{j=1}^{J}\mu_{j}-C\right\|}_{2}^{2}\right\}\right.\;$$

This sub-problem aims to update an image with respect to a fixed dictionary and associated sparse representation, which can be solved by the separate parabolic surrogate method [27]. Equation (9) can be iteratively solved as:

(10) $${\tilde{\mu }}_{j}^{k+1}=\mu_{j}^{k}-\frac{\sum_{i=1}^{I}\left(a_{ij}w_{i}\left({\left[A\mu^{k}\right]}_{i}-p_{i}\right)\right)+2\beta \sum_{s}\sum_{n=1}^{N}r_{nj}^{s}\left({\left[R_{s}\mu^{k}\right]}_{n}-{\left[D\alpha_{s}^{k+1}\right]}_{n}\right)+2\gamma (\sum_{j=1}^{J}{\tilde{\mu }}_{j}^{k}-C)}{\sum_{i=1}^{I}\left(a_{ij}w_{i}\sum_{m=1}^{J}a_{im}\right)+2\beta \sum_{s}\sum_{n=1}^{N}r_{nj}^{s}\sum_{m=1}^{J}r_{nm}^{s}+2\gamma }$$

where the superscript k=1,…,K represents the iteration number.

The implementation details of the proposed optimization algorithm are presented in Algorithm 1.
**Algorithm 1:** The outline of the optimization algorithm.1. 8 × 8 size image blocks are extracted from selected CT images to construct training samples, then we train dictionary D using an online learning method and estimate the value of direct component C using projected data. 2. Given:ε>0,β>0,γ>0,C, K. 3. Initialize: $\mu^{0}=0$$\alpha^{0}=0$. 4. Repeat: 5. Update the $\alpha^{k+1}$ using (8) and LASSO algorithm. 6. Update the reconstructed image $\mu^{k+1}$ using (10). 7. Until: the stop criteria are met. 8. Output:$\mu^{K}$

## 3. Experiments and Results

To verify the proposed reconstruction performance, the simulated and real CT data experimental results of the proposed algorithm are presented in this section. Meanwhile, the experimental results are compared to the SIR algorithm based on TV minimization without object support constraints (denoted as SIRTV + BOS); the SIR algorithm based on TV minimization with object support [18] (denoted as: SIRTV + OS); results of the SIR based on dictionary learning without object support (SIRDL + BOS) and the SIR algorithm based on dictionary learning with object support constraints [19] (SIRDL + OS). 

### 3.1. Human Chest Simulation

#### 3.1.1. The Simulated Low-Dose Projection

**Figure 1:**
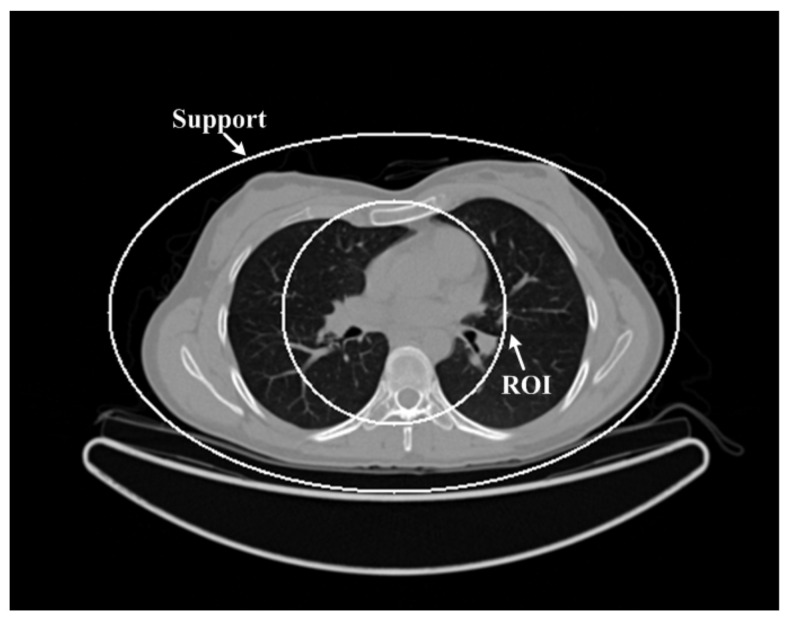
The chest image. The display window is [−1000 900]HU.

A human chest image [28] was downloaded from the website (http://wiki.kek.jp/pages/viewpage.action?pageId=13667347 accessed on 10 May 2021) to verify the performance of the above five different reconstruction algorithms. In this study, we used the downloaded chest image, which is given in Figure 1, as the ground truth. The size of the chest CT image was 512 × 512 pixels and the real size of each pixel was 0.9766 mm × 0.9766 mm. The equi-angular fan-beam uniformly collected the projection data of 360 views that only passed through the ROI region under the circular trajectory of 360°. The distances from the X-ray source to the rotation center and the detector were 570 mm and 1140 mm, respectively. In order to simulate low-dose projection data, Poisson noise with the photon numbers of *N_p_* = 1 × 10^5^, *N_p_* = 5 × 10^4^ and *N_p_* = 1 × 10^4^ was added to the ideal projection data to obtain three groups of low-dose projection data. The chest image, object support and ROI are shown in Figure 1.

#### 3.1.2. Global Dictionary Learning

**Figure 2:**
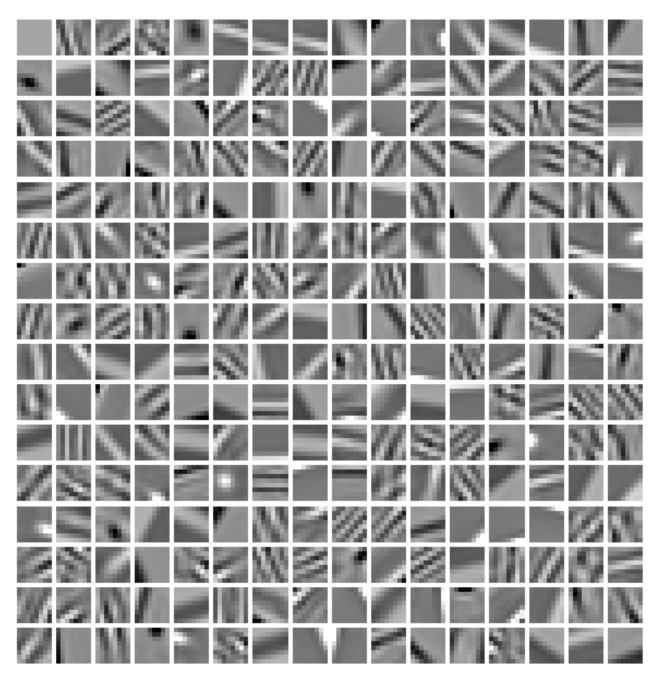
The dictionary trained using the chest image in Figure 1.

Image blocks with a size of 8 × 8 were firstly extracted from the chest CT image as training samples; then, image blocks with very small variance were removed. Finally, an online learning method was used to train the dictionary containing 256 atoms. The trained dictionary is shown in Figure 2.

#### 3.1.3. Reconstruction Results

**Figure 3:**
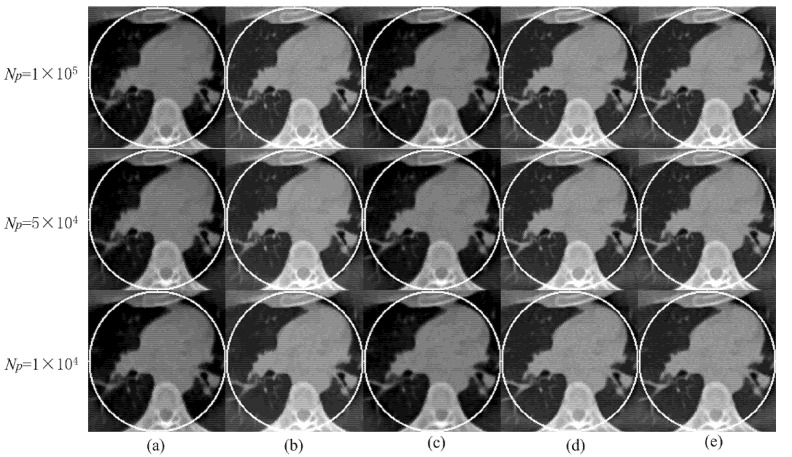
ROIs are reconstructed from three different noise levels using five different methods: (**a**) SIRTV + BOS; (**b**) SIRTV + OS; (**c**) SIRDL + BOS; (**d**) SIRDL + OS; (**e**) SIRDL + HL. The display window is [−1000 900]HU.

Using the fan-beam HLCC, we obtained the value of DC as 2.3328 × 10^3^.The sparse coding error of the proposed algorithm was set as ε = 1 × 10^7^; the maximum number of iterations was K = 50; number of ordered subsets was 40; and parameters β under the different low-dose projections of 1 × 10^5^, 5 × 10^4^ and 1 × 10^4^; the Poisson noise was set as 0.05, 0.08 and 1.5, respectively. For the parameters γ under different low-dose projections of 1 × 10^5^, 5 × 10^4^ and 1 × 10^4^, the Poisson noise was set as 1.13 × 10^−7^, 1.18 × 10^−7^ and 1.20 × 10^−7^, respectively. The reconstruction ROI of the five different methods under different noise levels is shown in Figure 3. It can be seen that both the SIRTV + BOS and SIRDL + BOS results have an obvious DC artifact. The DC artifact of the SIRTV + OS and SIRDL + OS results is very small, but the proposed algorithm in this paper basically eliminated the DC artifact. In addition, when the noise increased gradually, there were obvious block artifacts in the reconstruction result of the TV constraint, while the reconstruction algorithm based on the dictionary learning constraint eliminated the block artifacts and retained fine structures.

#### 3.1.4. Quantitative Analysis

In order to quantitatively evaluate the reconstruction performance of the proposed algorithm, we used two classical evaluation indexes to evaluate the experimental results; RMSE and SSIM were used to quantitatively measure the reconstruction algorithms:

(11) $$\mathrm{RMSE}=\frac{{\left\|\mu -\mu^{*}\right\|}_{2}}{\sqrt{N_{R}}}$$

(12) $$\mathrm{SSIM}=\frac{\left(2\bar{\mu }{\bar{\mu }}^{*}+c_{1})(2\sigma_{\mu \mu^{*}}+c_{2}\right)}{\left({\bar{\mu }}^{2}-{\bar{\mu }}^{*}{}^{2}+c_{1})(\sigma_{\mu }^{2}+\sigma_{\mu^{*}}^{2}+c_{2}\right)}$$

where μ is the reconstructed ROI; $\mu^{*}$ is the golden reference ROI; $N_{R}$ is the number of pixels in the reconstructed ROI; $\bar{\mu }$ and ${\bar{\mu }}^{*}$ are the mean value of μ and $\mu_{}^{*}$; $\sigma_{\mu }^{}$ and $\sigma_{\mu^{*}}^{}$ are the variation of μ and $\mu_{}^{*}$; $\sigma_{\mu \mu^{*}}$ is the covariance of μ and $\mu_{}^{*}$ and $c_{1}$ and $c_{1}$ are constants that we chose according to the reference [29].

Table 1 lists the RMSE and SSIM of the reconstruction results by different methods. It can be seen that the SIRTV + BOS algorithm has the maximum RMSE value and minimum SSIM value, whereas the SIRTV + HL algorithm has the minimum RMSE value and maximum SSIM value, indicating that the reconstruction quality of the proposed method is the highest.

### 3.2. Real CT Projection

**Figure 4:**
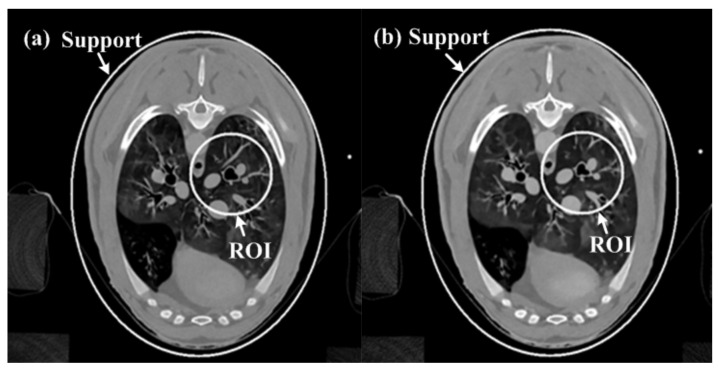
Reconstructed images from real CT projection data: (**a**) Normal dose; (**b**) low dose. The display window is [−700 800]HU.

A sheep chest was scanned with both normal (100 kV, 150 mAs) and low-dose (80 kV, 17 mAs) protocols on a SIEMENS Somatom Sensation 64-Slice CT Scanner using a circular cone-beam scanning mode. The sinogram of the central slices was extracted, which is fan-beam geometry. The radius of the trajectory was 57 cm. The 1160 projections were uniformly collected over a 360° range. For each projection, 672 detector elements were equi-angularly distributed to define a field of view (FOV) of 50.1 cm in diameter. We first reconstructed the global data of a goat lung at normal dose and low dose using the algorithm in the literature [19]; the reconstructed image was 768 × 768 pixels, the actual size was 43.63 cm × 43.63 cm, and we used this reconstructed image as the reference image. Then, we selected the area with a 61 pixels radius as the ROI. Finally, only the projection data through the ROI were kept, and the truncated projection data were obtained only through the ROI. The selected ROI is shown in Figure 4.

**Figure 5:**
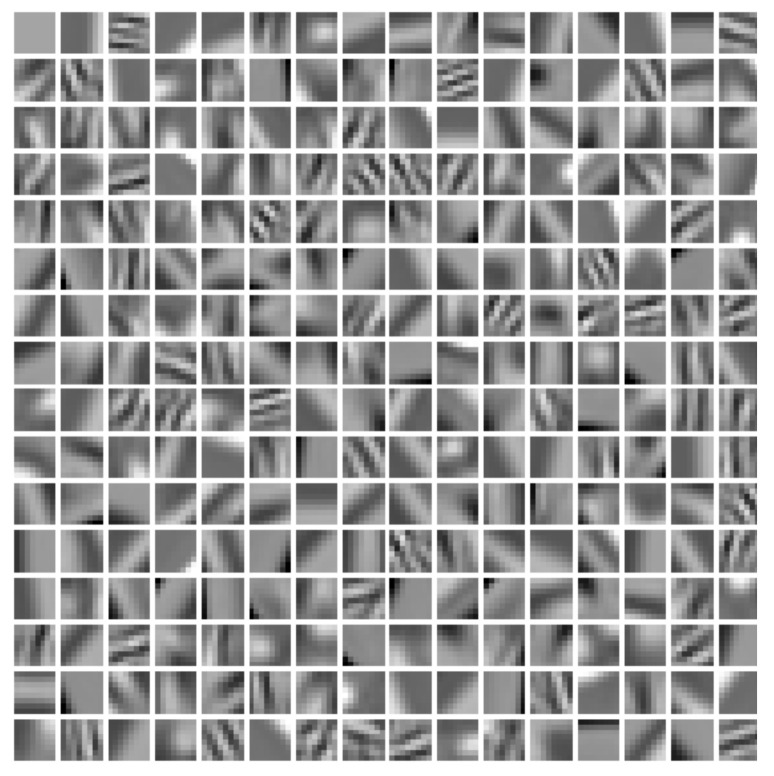
The dictionary trained using Figure 4.

To obtain a high-quality dictionary, an image block of size 8 × 8 pixels was extracted from the lung region of Figure 4a as a training sample in this section, and then a dictionary containing 256 atoms was trained using the dictionary learning method described in Section 3.1.2, as shown in Figure 5.

**Figure 6:**
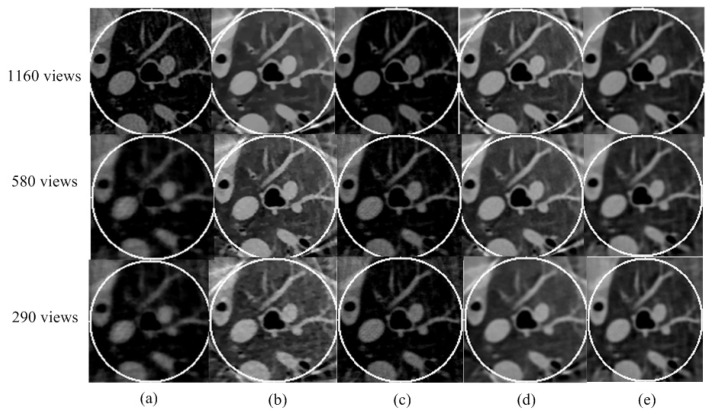
Reconstructed ROIs from normal-dose real sheep data using five different methods: (**a**) SIRTV + BOS; (**b**) SIRTV + OS; (**c**) SIRDL + BOS; (**d**) SIRDL + OS; (**e**) SIRDL + HL. The display window is [−700 800]HU.

**Figure 7:**
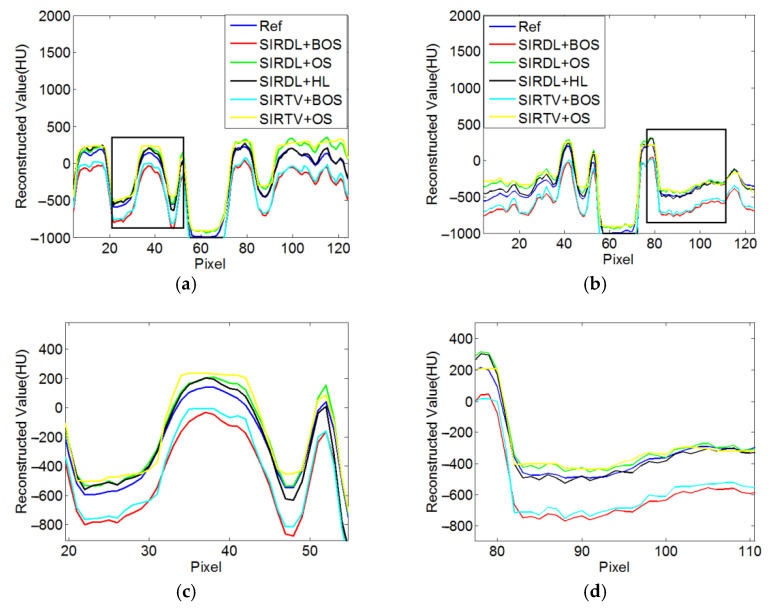
Profiles plots of ROIs reconstructed from 1160 views in Figure 6: (**a**) Horizontal central line; (**b**) vertical central line; (**c**) local enlargement of rectangle in (**a**); (**d**) local enlargement of rectangle in (**b**).

**Figure 8:**
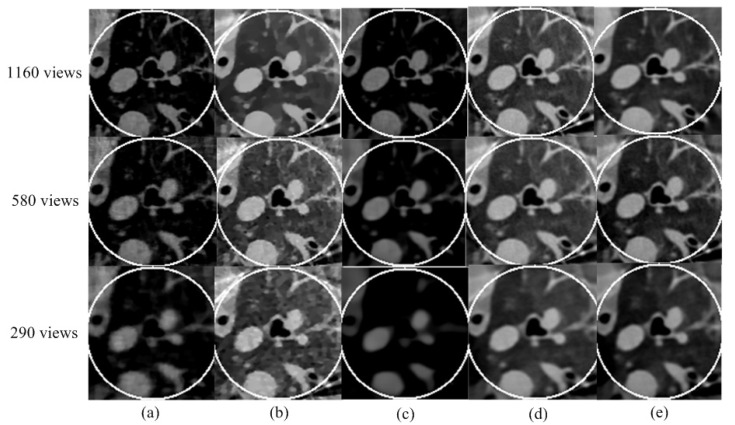
Reconstructed ROIs from low-dose real sheep projection data using five different methods:(**a**) SIRTV + BOS; (**b**) SIRTV + OS; (**c**) SIRDL + BOS; (**d**) SIRDL + OS; (**e**) SIRDL + HL. The display window is [−700 800]HU.

**Figure 9:**
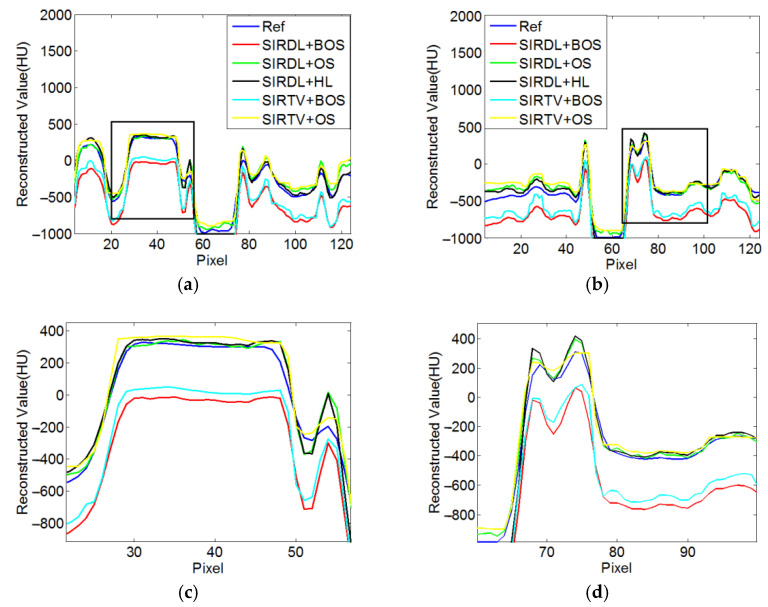
Profile plots of ROIs reconstructed from 1160 views in Figure 8: (**a**) Horizontal central line; (**b**) vertical central line; (**c**) local enlargement of rectangle in (**a**); (**d**) local enlargement of rectangle in (**b**).

According to the equal-angle fan-beam HLCC, the DCs of a normal dose and low dose were calculated as 2.9965 × 10^3^ and 3.1623 × 10^3^, respectively, and the parameters of the SIRDL + HL algorithm were set, as shown in Table 2. The reconstruction results of the five different algorithms for normal dose are shown in Figure 6. The representative profiles along the horizontal and vertical lines are shown in Figure 7. The above experiments were repeated with a low-dose sinogram, and corresponding results are shown in Figure 8 and Figure 9. The experimental results show that the image support constraint has a strong influence on the TV-based interior reconstruction method, and the reconstruction results of the SIRDL + HL algorithm basically eliminate the DC offset artifacts. The reconstructed image quality based on the TV constraint decreased significantly when the number of view angles was 580, while the reconstructed image quality of the SIRDL + HL method was basically the same as that of the full-view data. The reconstructed image quality based on the TV constraint had serious block artifacts when the number of view angles was 290, while the reconstructed image quality of this algorithm could obtain high reconstruction image quality.

**Figure 10:**
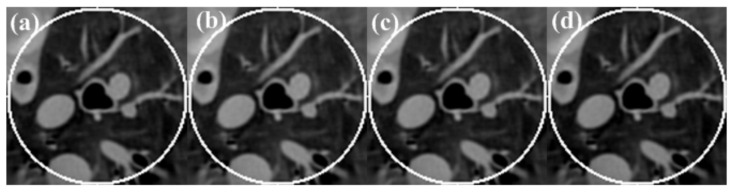
Reconstruction results with different DC: (**a**) DC = 2900; (**b**) DC = 3000; (**c**) DC = 3100; (**d**) DC = 3200. The display window is [−700 800]HU.

**Figure 11:**
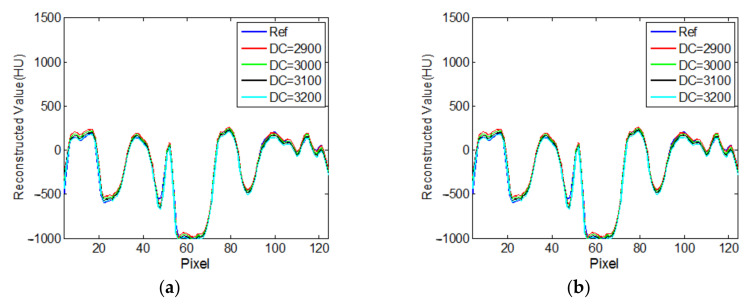
Profiles along different reconstruction results in Figure 10: (**a**) horizontal central line; (**b**) vertical central line.

To investigate the effect on the accuracy of an estimated DC value on the recovery of an intensity drop, the reconstructed ROIs from the normal-dose sinogram with four different DC values near the true value are shown in Figure 10. The corresponding central horizontal and vertical profiles are shown in Figure 11. It can be seen that the proposed algorithm is robust with respect to the DC value.

## 4. Discussion and Conclusions

Mathematically, there is no unique solution to the interior problem in CT reconstruction, and the reconstructed ROI image exhibits the DC shifts. Based on the assumption of a constant in the ROI region, TV-based interior tomography can eliminate the above artifacts well; however, in practical applications, the above assumptions do not necessarily hold, so TV constraint-based interior tomography will still show a slight DC artifact under the tight object support constraint. As early as 1992, the mathematician Maass had investigated the singular value decomposition of the Radon transform under the 2D interior problem and pointed out that the ROI reconstruction was almost exact except for a smooth unknown bias function, which is an approximately constant low-frequency component. Therefore, the difference between the DC value of the reconstructed image during the iterative process and the DC value calculated beforehand as a new constraint can eliminate the DC offset artifacts well.

Since dictionary sparse representation based on image blocks can effectively preserve the local structure information of images while suppressing image noise, introducing dictionary-based sparse representation into the statistical iterative reconstruction framework can effectively solve the interior problem, especially in a low-dose case. Extensive comparison of the experimental results shows that the performance of the dictionary learning-based interior tomography is better than that of the TV-based interior tomography.

The DC value of the cross-section where the ROI region is located needed to be calculated. In this paper, we used the untruncated projection data to calculate the value. Figure 10 and Figure 11 illustrate that the DC value of the cross-section image has little effect on the reconstruction results. In fact, we can use the truncated data to estimate the DC value. For example, we can estimate the ratio of the area of a cross-section of the human body to the area of the organ of interest; then, we can estimate the DC value of the whole cross-section using the DC value of the projection through the ROI region and the above ratio. The next step is to use the truncated projection data to estimate the DC component of the image of the cross-section where the ROI region is located.

In conclusion, we have incorporated sparse representation in terms of a learned dictionary and the constraint in terms of an image DC value into the SIR framework for interior tomography. Experimental results have demonstrated that our proposed approach reduces the DC artifact effectively and the DL-based algorithm outperforms the TV-based method in preserving fine structures, especially in a low-dose situation.

## Figures and Tables

**Table 1:** RMSE (HU)/SSIM comparisons of the reconstructed ROI images from simulated projection using different algorithms.

Algorithm	1 × 10^5^		5 × 10^4^		1 × 10^4^	
	RMSE	SSIM	RMSE	SSIM	RMSE	SSIM
SIRTV + BOS	258.2	0.3617	258.4	0.3629	259.4	0.3482
SIRTV + OS	139.2	0.6971	139.6	0.6829	142.3	0.6076
SIRDL + BOS	200.0	0.3903	201.1	0.3861	205.0	0.3746
SIRDL + OS	131.3	0.7108	132.3	0.6913	134.3	0.6238
SIRDL + HL	128.6	0.7204	134.3	0.7101	127.2	0.7021

**Table 2:** Selection of parameters of SIRDL + HL algorithm for normal (low)-dose sheep lung data.

The Number of View	ε	β	γ	The Number of Subset	The Number of Iteration
1160	$6.25\times 10^{-5}$	0.015(0.020)	$1.27\times 10^{-7}$	40	50
580	$6.25\times 10^{-5}$	0.022(0.030)	$8.00\times 10^{-8}$	20	80
290	$6.25\times 10^{-5}$	0.033(0.060)	$4.00\times 10^{-8}$	10	150

## Data Availability

The projection dataset of sheep lung is protected under the laws of our institution; hence, it is not open to the public.
